# Divergent Roles of Canonical and Non-Canonical Mismatch Repair in Regulating Temozolomide Sensitivity in Glioblastoma

**DOI:** 10.3390/ijms27146517

**Published:** 2026-07-22

**Authors:** Shiv K. Gupta, Sonia Jain, Teddy R. Friedman, Jann N. Sarkaria

**Affiliations:** Department of Radiation Oncology, Mayo Clinic, Rochester, MN 55905, USA

**Keywords:** MSH6, translesion synthesis, MGMT promoter methylation, replication stress, genomic instability, hypermutation, synthetic lethality, DNA damage response

## Abstract

Temozolomide (TMZ) remains the cornerstone of chemotherapeutic agent for glioblastoma (GBM), yet intrinsic and acquired resistance severely limits its clinical benefit. While O^6^-methylguanine-DNA methyltransferase (MGMT)–mediated repair of TMZ-induced O6-methylguanine (O^6^-meG) lesions has been extensively studied, the DNA mismatch repair (MMR) pathway is increasingly recognized as a key determinant of TMZ cytotoxicity. Canonical MMR, mediated by MutSα (MSH2–MSH6) and MutLα (MLH1–PMS2) complexes, recognizes O^6^-meG: thymine mispairs generated during replication and initiates futile repair cycles that culminate in replication stress, replication fork collapse, and apoptotic signaling; intact canonical MMR is, therefore, required for TMZ-induced cell death. Disruption of canonical MMR, frequently via acquired MSH6 mutations, confers TMZ tolerance and drives hypermutated recurrent GBM. Beyond mismatch correction, MMR proteins perform non-canonical functions in DNA damage signaling, replication stress responses, transcriptional regulation, chromatin dynamics, and immune modulation. These activities may shift the outcome from cytotoxic futile repair toward replication stress adaptation, Translesion synthesis (TLS)-mediated lesion tolerance, immune remodeling, and therapeutic resistance. Notably, partial attenuation or functional diversion of MMR may decouple lesion recognition from cytotoxic signaling, enabling TLS-mediated lesion tolerance without complete loss of MMR activity. This review integrates current insights into canonical and non-canonical MMR functions in GBM, defines their distinct contributions to TMZ sensitivity and resistance, and highlights therapeutic opportunities to exploit MMR-associated dependencies, including synthetic lethal strategies and immunotherapeutic vulnerabilities linked to MMR deficiency-driven hypermutation.

## 1. Introduction

Glioblastoma (GBM) is the most aggressive primary malignancy of the central nervous system, characterized by profound intratumoral heterogeneity, therapeutic resistance, and near-universal recurrence [1,2]. The current standard of care—maximal surgical resection followed by radiotherapy and concurrent and adjuvant (i.e., during radiotherapy and after radiotherapy, respectively) temozolomide (TMZ) chemotherapy, yields a median overall survival of approximately 14–16 months [3,4]. TMZ, an oral alkylating agent capable of crossing the blood–brain barrier, exerts its antitumor activity primarily through induction of cytotoxic DNA lesions [5]. However, the therapeutic efficacy of TMZ is highly variable, and resistance develops in nearly all patients, underscoring the importance of tumor-intrinsic DNA repair and metabolic pathways—the latter influencing nucleotide availability, replication stress, and damage tolerance—in shaping treatment response [6,7].

TMZ induces DNA adducts at multiple sites, including N^7^-methylguanine (N^7^-meG) and N^3^-methyladenine (N^3^-meA) repaired by base excision repair (BER), as well as the less frequent but highly cytotoxic O^6^-methylguanine (O^6^-meG) lesion, which is reversed by O^6^-methylguanine-DNA methyltransferase (MGMT) [5]. MGMT is epigenetically silenced in ~40% of GBM cases, and its promoter methylation is associated with improved therapeutic response and survival, establishing MGMT as a primary determinant of TMZ sensitivity [8]. However, this biomarker does not fully explain clinical resistance, as a substantial fraction of MGMT-methylated GBMs exhibit intrinsic or acquired resistance, implicating additional modulators of TMZ efficacy [9,10]. Among these, the mismatch repair (MMR) pathway plays a critical yet underappreciated role. Although MMR normally preserves genomic integrity by correcting base mismatches, in the context of TMZ treatment it paradoxically mediates cytotoxicity through recognizing O^6^-meG:Thymidine (O^6^-meG:T) mispairs, triggering futile cycles of excision and resynthesis that ultimately generate replication-associated double-strand breaks, replication fork collapse, and apoptotic cell death [11,12]. Thus, intact MMR is essential for TMZ-induced cell killing.

Conversely, loss of canonical MMR function, most commonly through mutations or downregulation of MSH6, or other core MMR components in recurrent GBM, abrogates mismatch recognition and permits replication across O^6^-meG lesions [13,14,15]. This tolerance enables tumor cell survival under TMZ pressure, at the cost of dramatically increased mutational burden [16,17]. Clinically, this manifests as a hypermutator phenotype enriched in TMZ signature mutations, driving clonal evolution, therapeutic escape, and disease progression [16,17,18]. Importantly, MMR-deficient GBMs often retain MGMT promoter methylation, highlighting the independent and cooperative roles of these repair pathways in modulating TMZ sensitivity and resistance [19,20].

Beyond their canonical roles in mismatch recognition and repair, MMR proteins participate in a growing array of non-canonical processes [21,22]. Emerging evidence indicates that MMR proteins exert broader non-canonical functions beyond mismatch excision repair, including direct participation in DNA damage signaling, regulation of replication stress responses, coordination with BER and HR pathways, and modulation of transcriptional and chromatin-associated programs [23,24,25,26,27]. Such non-canonical MMR activities may be particularly relevant to the evolution of TMZ resistance. In this setting, partial loss of MMR function—arising from functional diversion, altered stoichiometry, or post-translational modification of MMR components—can rewire DNA damage responses (DDR). Although this area remains underexplored, these contexts may enable GBM cells to evade TMZ-induced apoptosis while maintaining sufficient repair capacity to tolerate ongoing replicative stress.

Collectively, these observations position the MMR pathway, both canonical and non-canonical, as a central modulator of TMZ sensitivity and resistance in GBM. Understanding how distinct MMR functions are engaged, suppressed, or repurposed during therapy is essential for refining predictive biomarkers, designing rational combination therapies, and exploiting emergent vulnerabilities such as synthetic lethality within DNA repair networks, and hypermutation-associated immunogenicity. Beyond conventional DNA-damaging approaches, emerging therapeutic strategies for GBM—including novel combination regimens, radiotherapy optimization, and investigational approaches such as phytotherapy and mycotherapy that may modulate redox homeostasis, ferroptosis, and DDR [28,29,30]—represent an expanding therapeutic landscape within which MMR-dependent mechanisms operate. This review explores the mechanistic basis and clinical relevance of MMR-mediated TMZ resistance, with an emphasis on translating pathway insights into therapeutic opportunity.

## 2. TMZ-Induced DNA Methyl Adducts and Repair Pathways

**Figure 1:**
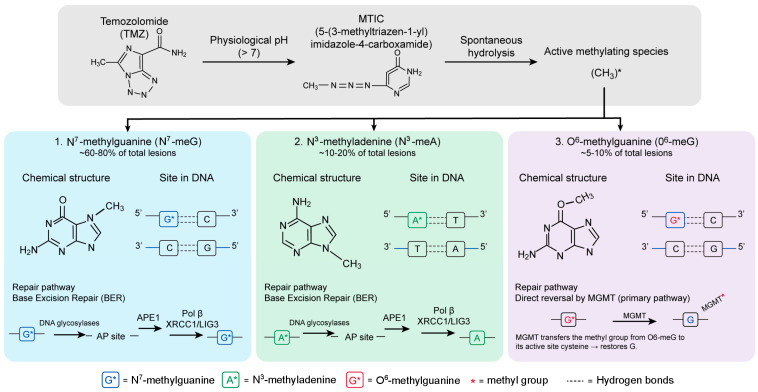
**Major DNA lesions induced by TMZ, and their principal repair pathways.** TMZ generates three predominant DNA adducts: N^7^-meG, N^3^-meA, and O^6^-meG. N^7^-meG and N^3^-meA are primarily repaired by BER, whereas O^6^-meG is repaired by direct reversal through MGMT. Lesion frequencies are approximate and may vary depending on TMZ dose, cell type, and experimental conditions.

TMZ induces cytotoxic DNA lesions (Figure 1) through spontaneous hydrolysis to the active intermediate 5-(3-methyltriazen-1-yl)-imidazole-4-carboxamide (MTIC), which subsequently generates methyldiazonium ion that transfers methyl groups to DNA bases [31]. The three principal methylation adducts are O^6^-meG, N^7^-meG, and N^3^-meA. Among these, N^7^-meG (~60–80%) and N^3^-meA (~10–20%) are the most abundant lesions and are primarily repaired through the BER pathway (Figure 1) [32,33]. BER-mediated repair involves lesion recognition by DNA glycosylases followed by endonuclease cleavage, gap filling by DNA polymerases, and DNA ligation, thereby restoring genomic integrity and promoting cell survival, which contributes to intrinsic resistance to TMZ [34]. However, the role of BER in determining TMZ response is highly context-dependent. Under conditions of extensive alkylation damage or when BER intermediates accumulate more rapidly than they can be resolved, BER-generated ssDNA repair intermediates may become cytotoxic, particularly when PARP-dependent repair is impaired or overwhelmed. In this setting, persistent ssDNA gaps and PARP-trapping lesions can stall replication forks, exacerbate replication stress, and ultimately promote cell death [35,36,37]. BER is especially critical in post-mitotic cells, such as neurons, where it maintains genome integrity and prevents transcriptional blockade throughout their long lifespan [38,39]. Conversely, in rapidly proliferating tumor cells, excessive BER engagement following TMZ treatment may paradoxically enhance replication-associated cytotoxicity by increasing the burden of unresolved repair intermediates [35,37].

**Figure 2:**
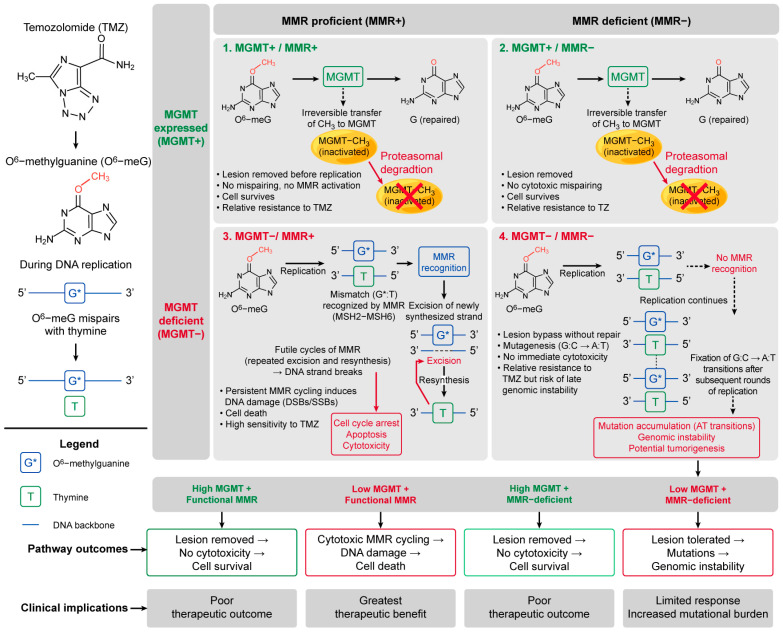
**Canonical processing of O^6^-meG: a binary model governed by MGMT and MMR status.** In cells expressing MGMT, regardless of MMR status (MGMT^+^/MMR^+^ or MGMT^+^/MMR^−^; panels 1 and 2), O^6^-meG lesions are efficiently repaired by direct reversal. During this reaction, MGMT acts as a suicide enzyme, transferring the methyl group from O^6^-meG to its active-site cysteine residue and subsequently undergoing proteasomal degradation, thereby preventing lesion persistence and cytotoxicity. In contrast, when MGMT is absent, silenced, or saturated (MGMT^−^/MMR^+^; panel 3), O^6^-meG persists into S phase and mispairs with thymine. These O^6^-meG mismatches are recognized by the MMR complexes, triggering futile cycles of mismatch repair that fail to remove the O^6^-meG adduct on the template strand. Repeated rounds of excision and resynthesis generate replication stress, ATR-CHK1 signaling, and ultimately apoptosis. Conversely, MMR deficiency (MGMT^−^/MMR^−^; panel 4) prevents mismatch recognition, allowing lesion tolerance with accumulation of G→A transition mutations, and therapeutic resistance. Together, these four molecular scenarios illustrate how the interplay between MGMT and MMR status determines whether TMZ-induced O^6^-meG lesions are accurately repaired, converted into cytotoxic DNA damage, or tolerated through mutagenic lesion bypass, thereby dictating therapeutic response. Dashed arrows indicate proposed alternative mechanisms.

In contrast, O^6^-meG represents a minor (~5–10%) but highly cytotoxic lesion driving replicative stress and DNA damage (Figure 1) [32,33]. This lesion can be directly reversed by the DNA repair protein MGMT through a suicide reaction that transfers the methyl group from O^6^-meG to an internal cysteine residue in the enzyme, thus directly repairing the lesion [40]. High MGMT expression, therefore, confers primary resistance to TMZ by repairing O^6^-meG lesions before DNA replication occurs. When O^6^-meG lesions persist due to low MGMT activity, they mispair with thymine (T) during DNA replication, generating O^6^-meG:T mismatches that are recognized by the MMR machinery (Figure 2) [41,42]. Instead of correcting the lesion, MMR repeatedly excises thymine from the nascent DNA while leaving the O^6^-meG adduct intact, resulting in futile cycling of MMR. This iterative process triggers replication stress, accumulation of ssDNA gaps, and ultimately DNA double-strand breaks (DSBs) during subsequent replication cycles [23,32]. Activation of DNA damage signaling pathways promotes cell cycle arrest and apoptosis, leading to TMZ sensitivity in actively dividing cells [43].

Loss or inactivation of MMR components, including MLH1, MSH2, or MSH6, disrupts recognition and processing of O^6^-meG:T mismatches (Figure 2), thereby preventing the futile repair cycling normally associated with canonical MMR signaling [21,25,44]. As a result, cells tolerate O^6^-meG lesions without triggering cytotoxic signaling, leading to acquired TMZ resistance and increased mutagenesis. Thus, the balance between MGMT-mediated direct repair, BER processing of abundant methylated bases, and MMR-dependent cytotoxic signaling ultimately determines whether TMZ exposure results in cell killing or resistance [45].

## 3. TMZ as a Biomarker-Guided Therapy in GBM

TMZ remains the cornerstone chemotherapeutic agent in the standard-of-care treatment for GBM, as established by the Stupp protocol [4]. This regimen— maximal safe surgical resection followed by concurrent radiotherapy with daily TMZ and subsequent adjuvant TMZ cycles—significantly improves overall survival compared with radiotherapy alone [3]. The therapeutic efficacy of TMZ is critically influenced by MGMT, which repairs the cytotoxic O^6^-meG lesions induced by TMZ [8,23,32]. Consequently, MGMT promoter methylation—leading to epigenetic silencing—has emerged as a clinically validated predictive biomarker in GBM, reflecting impaired DNA repair capacity and enhanced TMZ sensitivity. Approximately 40% of newly diagnosed GBMs harbor MGMT promoter methylation, allowing persistence of O^6^-meG lesions and increased therapeutic response to TMZ [8].

Despite this strong biological rationale, the influence of MGMT promoter methylation on therapeutic decision-making varies between the United States and Europe [46,47]. In the U.S., MGMT status is routinely assessed for prognostic evaluation but has limited impact on first-line treatment in younger, fit patients, who receive combined radiotherapy and TMZ irrespective of methylation; its utility is greatest in elderly or frail populations, where it informs the choice between TMZ-based therapy and radiotherapy alone. In contrast, European practice, guided by the European Association of Neuro-Oncology, more explicitly integrates MGMT status into treatment algorithms: MGMT-methylated tumors are preferentially treated with TMZ-containing regimens, whereas unmethylated tumors are managed with radiotherapy-based approaches to avoid unnecessary chemotherapy toxicity. This divergence reflects broader philosophical differences, with U.S. practice favoring broader treatment application and European approaches emphasizing biomarker-driven therapeutic selection.

Notwithstanding its clinical utility, MGMT promoter methylation has important limitations as a predictive biomarker [48]. Intratumoral heterogeneity can result in variable MGMT levels across different tumor regions, and promoter methylation does not always correlate with MGMT protein levels or enzymatic activity due to additional transcriptional and post-transcriptional regulatory mechanisms [10,48,49,50]. Clinically, a substantial minority of MGMT-methylated GBM cases fail to respond to TMZ, while a subset of unmethylated tumors derives modest benefit; moreover, approximately 10% of cases fall within the “grey zone” of intermediate methylation, where classification is ambiguous and predictive value is uncertain [4,8,17,51]. Collectively, these limitations underscore the contribution of alternative regulatory mechanisms, DNA repair pathways, and resistance pathways, and highlight that MGMT promoter methylation alone is an imperfect biomarker and should be integrated with additional molecular determinants to more accurately predict TMZ responsiveness.

Emerging evidence suggests that even subtle alterations in MMR function, either by gene expression pattern or post-translational modifications, may drive therapeutic resistance in GBM [20,52]. Mechanistically, MMR capacity in GBM is tuned not only by inactivating mutations but also by transcriptional regulation of core components such as MSH2 and MSH6, and even modest reduction in their abundance is sufficient to confer TMZ resistance and predict initial treatment response [52,53]. Importantly, these quantitative deficits typically arise without detectable microsatellite instability (MSI) and are often missed by MMR-protein immunohistochemistry, while MGMT promoter methylation, the current standard, does not reliably reflect protein expression or repair activity [17,52,54]. Therefore, assessment of the functional integrity of the MMR pathway rather than any single genetic, epigenetic, or immunohistochemical surrogate, may serve as an important and independent determinant of TMZ response in GBM.

Collectively, these insights highlight the need to move beyond single-biomarker approaches toward integrated models that capture the dynamic activity of DNA repair networks to better inform therapeutic strategies.

## 4. Diverse Roles of MMR: Implications for TMZ Response

**Figure 3:**
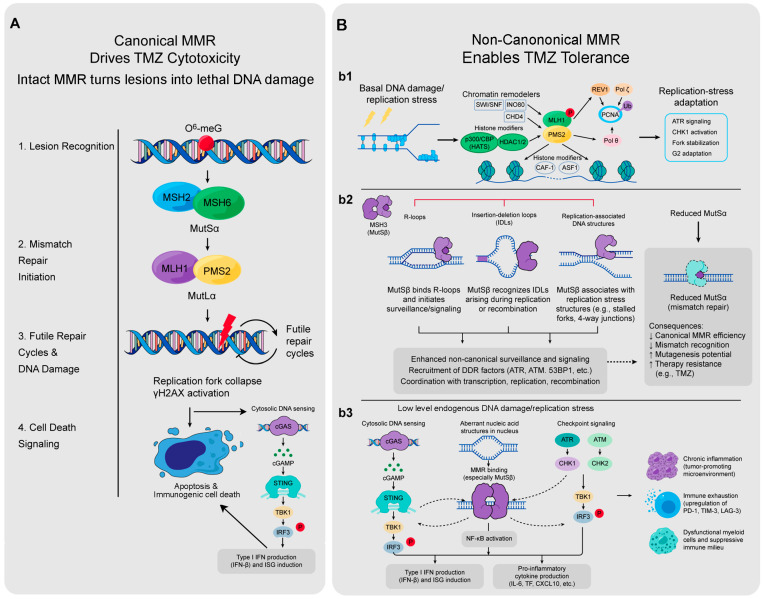
**Canonical and non-canonical MMR activities differentially regulate TMZ response in GBM**. (**A**) Canonical MMR promotes TMZ cytotoxicity. O^6^-meG mismatches generated during replication are recognized by MutSα and processed by MutLα, triggering futile repair cycles, ssDNA gap formation, replication stress, ATR-CHK1 activation, and DNA damage signaling that culminates in cell-cycle arrest, apoptosis, while cytosolic DNA sensing may initiate immunogenic cell death. (**B**) Non-canonical MMR activities promote adaptation and resistance. (**b1**) ATM-dependent MLH1 phosphorylation enables interactions with chromatin regulators and DDR factors, potentially shifting MMR outputs from apoptosis toward replication-stress adaptation and TLS-mediated lesion tolerance. (**b2**) Increased MutSβ (MSH2–MSH3) formation competes with MutSα for MSH2, reducing O^6^-meG recognition and cytotoxic signaling while favoring lesion tolerance and genomic instability. (**b3**) Persistent MMR engagement with low level endogenous DNA lesions activates cGAS–STING and ATR/ATM signaling, inducing interferon and inflammatory responses that may initially support anti-tumor immunity but, when chronic, promote immune suppression and therapeutic resistance. Dashed arrows indicate proposed or indirect interactions.

The MMR pathway exerts multifaceted control over genome stability, DNA damage signaling, and therapeutic response [25]. Traditionally, MMR has been viewed through the lens of its canonical role in post-replicative mismatch correction [25]. However, increasing evidence indicates that MMR proteins also perform non-canonical functions that are mechanistically and functionally distinct from classical mismatch repair. In GBM, these two facets of MMR, canonical and non-canonical, have divergent and opposing effects on TMZ sensitivity and resistance (Figure 3).

### 4.1. Canonical MMR: A Driver of TMZ Cytotoxicity

Canonical MMR is executed through the coordinated actions of mismatch recognition complexes MutSα and MutSβ, together with the downstream repair effector MutLα (MLH1–PMS2) [25,55]. These complexes correct base–base mismatches and insertion–deletion loops arising during DNA replication, thereby maintaining genomic integrity [25,55]. During TMZ treatment, however, this pathway assumes a paradoxically pro-cytotoxic role. In MGMT-methylated tumors, epigenetic silencing of MGMT prevents removal of TMZ-induced O^6^-meG adducts, allowing these lesions to persist and mispair with thymine during replication [8]. The resulting O^6^-meG mismatches are recognized by MutSα and processed through MutLα-mediated excision [56]. Because MMR selectively removes the newly incorporated thymine while leaving the O^6^-meG lesion in the template strand unresolved, repeated cycles of excision and resynthesis—termed futile repair—are initiated [56,57]. These cycles generate persistent single-stranded DNA (ssDNA) gaps, activate ATR–CHK1 signaling, destabilize replication forks, and ultimately trigger replication fork collapse, double-strand break formation, cell-cycle arrest, and apoptosis [56,57]. Accordingly, intact canonical MMR is indispensable for TMZ-induced cytotoxicity (Figure 3), whereas disruption of key components, particularly MSH6, permits lesion tolerance and contributes to acquired TMZ resistance [13,15].

Despite the central role of MMR in mediating TMZ cytotoxicity, pathogenic alterations in core MMR genes are relatively infrequent in both primary and recurrent GBM [17]. Moreover, many hypermutated recurrent tumors lack identifiable MMR mutations, and classical MSI remains rare, indicating that complete MMR deficiency is unlikely to represent the predominant mechanism of TMZ resistance in MGMT-methylated GBM [17,19,52,53,58,59]. Instead, accumulating evidence supports a model of functional attenuation or pathway rewiring, whereby MMR activity is reduced without complete pathway inactivation. Such alterations may arise through transcriptional downregulation, post-translational modification of MMR proteins, altered MutSα/MutSβ stoichiometry that limits efficient mismatch recognition, or partial loss-of-function mutations [19,52,53,60]. Collectively, these changes may generate an MMR-hypomorphic state, characterized by diminished checkpoint activation and cytotoxic signaling while preserving sufficient repair, while attenuating checkpoint activation and cytotoxic signaling in response to TMZ-induced damage [19,52]. Because these functional alterations are not readily detected by conventional immunohistochemistry or MSI testing, MMR dysfunction in GBM is likely underrecognized [58,59]. Functional assays that directly quantify MMR activity, particularly plasmid-based reporter systems, may, therefore, provide a more accurate assessment of MMR competence [52]. Integration of such assays with CLIA-approved MGMT promoter methylation testing could ultimately improve biomarker-guided prediction of TMZ responsiveness by capturing functional repair capacity rather than protein expression alone [52,54].

### 4.2. Non-Canonical MMR: Facilitator of Stress Tolerance and Adaptive Resistance

Beyond post-replicative mismatch correction, MMR proteins participate in a growing spectrum of non-canonical functions that are independent of classical mismatch excision (Figure 3). These activities include regulation of DNA damage signaling, replication stress responses, DNA repair pathway choice, transcriptional regulation, chromatin organization, and immune signaling, and are increasingly recognized as important determinants of therapeutic response. In tumors with partial or context-dependent impairment of MMR, these non-canonical functions may become dominant, shifting the cellular response from cytotoxic DNA damage processing toward lesion tolerance, adaptive survival, and therapeutic resistance [17].

#### 4.2.1. DNA Damage Signaling and Replication Stress

Beyond their role in mismatch recognition and repair, MMR proteins directly interface with DNA damage signaling pathways. MSH2–MSH6 can function as a sensor of aberrant DNA structures, facilitating ATR activation and downstream checkpoint signaling independently of mismatch excision [56,61]. In GBM, where chronic replication stress is a hallmark of oncogenic signaling, these non-canonical MMR activities are likely to shape cellular responses to TMZ-induced DNA damage. As a result, the functional outcome of ATR-CHK1 activation in response to TMZ-induced DNA damage is context-dependent and reflects the intensity and duration of signaling. In MMR-proficient cells, futile repair cycles generate extensive ssDNA intermediates that elicit robust ATR–CHK1 activation, initially slowing DNA replication and promoting fork stabilization, but ultimately triggering replication fork collapse, double strand break formation, and apoptosis when the burden of DNA damage exceeds repair capacity [56,57,62]. By contrast, partial attenuation of MMR activity may reduce the accumulation of futile repair intermediates, thereby dampening checkpoint and apoptotic signaling while preserving sufficient lesion recognition to support replication fork stability and cell survival [52]. Consequently, MMR-hypomorphic cells may become increasingly dependent on ATR–CHK1 signaling to maintain replication fork integrity and regulate origin firing, promoting lesion tolerance rather than cytotoxicity [61,63,64,65]. This distinction—acute high-intensity checkpoint activation driving cell death versus chronic, moderate signaling supporting replication stress adaptation—may explain why ATR inhibition is synthetically lethal in MMR-deficient contexts while ATR activation contributes to TMZ cytotoxicity in MMR-proficient cells [65].

Constitutive ATM activation, reported in subsets of TP53-mutant GBMs [66], may further reprogram MMR-dependent signaling by enhancing replication fork stabilization, checkpoint adaptation, and lesion-tolerance pathways (Figure 3B). In this model, MMR complexes remain capable of recognizing O^6^-meG mismatches, yet downstream cytotoxic signaling is attenuated, shifting the balance from futile repair toward lesion tolerance. Throughout this review, we refer to this functional phenotype as an MMR-hypomorphic state, in which MMR activity is attenuated—but not completely abolished—through mechanisms such as reduced MMR expression, altered protein stoichiometry, post-translational modification, or partial loss-of-function mutations [19,52,58,59]. Despite their distinct origins, these alterations converge on a common phenotype characterized by attenuated checkpoint signaling, enhanced Translesion Synthesis (TLS)-mediated lesion tolerance, and preservation of sufficient repair activity to avoid the profound genomic instability associated with complete MMR deficiency [52,53]. Such a state may provide a mechanistic framework for understanding how GBM cells evade TMZ-induced cytotoxicity while retaining partial MMR function, thereby facilitating clonal evolution, adaptive resistance, and tumor recurrence [17,52].

#### 4.2.2. Crosstalk with Other DNA Repair Pathways

Crosstalk between the MMR and other DNA repair mechanisms is a key determinant of genome stability and therapeutic response in cancer. Beyond mismatch correction, MMR functions as a signaling hub that interfaces with the DDR to coordinate repair pathway choice under replication stress [25,61]. Persistent engagement of MMR at O^6^-meG lesions generates ssDNA intermediates that activate ATR–CHK1 signaling while limiting access to TLS polymerases to damaged DNA [57,67]. Consequently, intact MMR suppresses mutagenic lesion bypass, whereas MMR attenuation or loss favors TLS-dependent damage tolerance and mutagenesis [17,67]. MMR further cooperates with BER during alkylation damage processing and with Homologous Recombination (HR) to resolve replication-associated double-strand breaks while preserving recombination fidelity [36]. Under conditions of excessive DNA damage or HR deficiency, persistent MMR-driven replication stress may promote repair through Non-Homologous End Joining (NHEJ), thereby increasing genomic instability [68]. Emerging evidence also implicates MMR proteins in the resolution of transcription-associated structures, such as R-loops, further linking MMR to chromatin organization and transcriptional regulation [69]. Collectively, these findings position MMR as a central regulator of DNA repair pathway choice, balancing high-fidelity repair with lesion tolerance. Disruption of this balance promotes mutagenesis, genome instability, and therapeutic resistance [25].

#### 4.2.3. Transcriptional and Epigenetic Regulation

Beyond mismatch correction, the MMR pathway serves as a critical interface between genome maintenance, chromatin regulation, and transcriptional control. MMR activity is tightly coupled to chromatin context, with MutSα (MSH2–MSH6) preferentially recruited to H3K36me3-marked gene bodies through SETD2, thereby safeguarding transcriptionally active regions and maintaining transcriptional fidelity [70]. During genotoxic stress, persistent MMR engagement at replication-associated DNA lesions generates repair intermediates that activate ATR–CHK1 signaling, coupling lesion processing to replication checkpoint activation transcriptional reprogramming [56,61]. Although not a consequence of functional diversion of MMR pathway, in several human cancers, MMR deficiency, frequently resulting from MLH1 promoter hypermethylation, is associated with widespread epigenomic remodeling, including altered DNA methylation, histone modifications, and chromatin accessibility [71]. Together with an elevated mutational burden, these changes may reshape regulatory landscapes, alter transcription-factor binding, and rewire gene-expression programs, thereby promoting cellular plasticity and intratumoral heterogeneity [17]. Emerging evidence further suggests that MMR proteins contribute directly to transcriptional regulation through interactions with RNA polymerase II and the resolution of R-loop structures (Figure 3B) [69,72]. Collectively, these findings highlight MMR as a regulator of genomic stability as well as transcriptional and epigenetic homeostasis. In GBM, disruption or rewiring of these non-canonical MMR functions under TMZ-induced replication stress may contribute to transcriptional plasticity, acquisition of stem-like states, and therapeutic resistance [18,73].

Persistent non-canonical MMR signaling also imposes substantial bioenergetic and biosynthetic demands through sustained checkpoint activation, repair synthesis, and PARP1-dependent NAD^+^ consumption [74]. Rather than directly regulating metabolism, these functions are proposed to create a metabolic stress state that selects for adaptive rewiring of nucleotide biosynthesis, redox homeostasis, and energy metabolism to sustain DNA replication [75]. Once established, metabolic adaptation may influence MMR and TLS pathway choice by altering intracellular dNTP availability, modulating repair synthesis, and remodeling chromatin architecture, thereby affecting MMR factor recruitment and TLS engagement [6,76,77]. Although the mechanistic links remain incompletely understood, accumulating evidence supports a model in which metabolic adaptation, in part supported by MMR-associated transcriptional and epigenetic regulation, biases the cellular response toward TLS-mediated damage tolerance while limiting MMR-dependent cytotoxicity during TMZ treatment [67,78].

#### 4.2.4. Immune Modulation and Tumor Evolution

MMR machinery also exerts profound downstream effects on antitumor immunity and tumor evolution. The immune and evolutionary consequences of MMR activity in TMZ-treated GBM are best viewed as downstream effects of canonical MMR engagement or MMR deficiency rather than intrinsic non-canonical MMR functions. As discussed in Section 4.2.1, Section 4.2.2 and Section 4.2.3, non-canonical MMR activities encompass direct roles in checkpoint signaling, replication stress tolerance, chromatin regulation, and transcriptional control. In contrast, the immune signaling and tumor evolution discussed below arise secondarily from MMR-dependent DNA damage signaling or the genomic instability associated with impaired MMR. Although mechanistically distinct, these processes have implications for TMZ response.

During futile processing of O^6^-meG lesions, MMR generates persistent ssDNA intermediates that activate ATR–CHK1 signaling and promote replication stress [57,61]. Under these conditions, replication-associated DNA damage can result in cytosolic DNA accumulation and activation of the cGAS–STING pathway, inducing type I interferon signaling and enhancing tumor immunogenicity [79]. Consequently, MMR-proficient tumors can transiently acquire an immunostimulatory phenotype, that may increase susceptibility to immune-mediated clearance (Figure 3A) [79]. In contrast, MMR-deficient or MMR-hypomorphic tumors accumulate mutations while exhibiting diminished replication stress signaling and altered innate immune responses. In addition to hypermutation, these tumors frequently display epigenetic remodeling, including silencing of interferon-stimulated genes and rewiring of regulatory networks that promote immune checkpoint expression and myeloid-dominated, immunosuppressive tumor microenvironment (Figure 3B) [17,18]. These effects are particularly relevant in GBM, where STING expression is often reduced in tumor cells, partly through promoter methylation, thereby further limiting innate immune activation despite increased mutational burden [80]. Concurrently, loss of sustained MMR engagement shifts the balance from cytotoxic signaling toward lesion tolerance, fostering clonal evolution and selection of variants with altered antigen presentation, interferon responsiveness, and metabolic programs that may support immune escape [17,18,73]. Thus, repeated TMZ exposure can drive tumor evolution by coupling MMR-dependent genomic diversification with epigenetic and immune remodeling, ultimately shaping therapeutic sensitivity and resistance.

Collectively, these observations underscore the multifaceted role of MMR in determining TMZ response. Canonical MMR promotes tumor cell killing through persistent processing of O^6^-meG lesions, whereas impaired or rewired MMR signaling facilitates genomic evolution and adaptive resistance [8,17]. Defining how these interconnected processes influence immune surveillance and therapeutic response may reveal new opportunities to overcome TMZ resistance and improve therapeutic response in GBM.

## 5. Integration of MMR with TLS: Mechanism of Stress Tolerance

TLS is a DNA damage tolerance pathway that enables replication across damaged DNA templates, allowing cells to bypass lesions at the cost of reduced genomic fidelity [81,82]. In GBM, emerging evidence suggests that the MMR machinery may influence TLS-mediated bypass of TMZ–induced DNA lesions [67]. However, the mechanistic relationship between MMR and TLS remains poorly defined [67,83]. Current observations primarily implicate non-canonical MMR functions in facilitating TLS activity, potentially promoting lesion bypass and mutagenic tolerance under therapeutic stress [83]. In contrast, the role of canonical MMR in modulating TLS has not been clearly established [83,84]. Elucidating how canonical and non-canonical MMR pathways intersect with TLS may provide important insights into adaptive DNA damage tolerance mechanisms that contribute to TMZ resistance in GBM [67].

### 5.1. Canonical MMR as a Proposed Suppressor of TLS

In MMR-proficient cells, a prevailing model proposes that the repair machinery may suppress TLS through coordinated control of lesion processing, replication checkpoint signaling, and polymerase access to damaged DNA [83,84]. Following TMZ exposure, O^6^-meG mismatches are recognized by MutSα, which recruits MutLα (MLH1–PMS2) to initiate excision and generate replication protein A (RPA)-coated ssDNA gaps during futile repair cycling [25,55,57]. Importantly, O^6^-meG itself is not a strong replication-blocking lesion [85,86]. Instead, the primary substrate for TLS in this setting comprises the ssDNA gaps generated as secondary intermediates of MMR processing rather than the O^6^-meG adduct itself [67]. Concurrently, MMR-dependent activation of the ATR–CHK1 pathway slows replication fork progression and is proposed to restrain PCNA monoubiquitination, thereby limiting recruitment of TLS polymerases [56,61,64,87]. Repeated cycles of futile repair may further create a kinetic barrier that continuously re-engages the lesion, favoring repair-associated DNA synthesis over mutagenic bypass [57,84]. Collectively, these mechanisms are proposed to establish a checkpoint-enforced, repair-dominant state that channels O^6^-meG lesions toward replication stress and cytotoxic signaling rather than damage tolerance [56,61].

By contrast, attenuation or loss of MMR relieves these constraints, permitting RAD18-dependent PCNA monoubiquitination and more efficient TLS-mediated lesion bypass, thereby promoting DNA damage tolerance, hypermutation, and therapeutic resistance [67,83]. However, the mechanistic relationship between MMR and TLS is likely more nuanced than a simple antagonistic model. Recent studies suggest that, under specific contexts, MMR proteins may also facilitate TLS polymerase recruitment and post-replicative gap filling, indicating that MMR can either suppress or promote TLS depending on the nature of the DNA lesion, the stage of lesion processing, and the cellular context [67,83,84]. Collectively, these observations support a model in which canonical MMR functions as an anti-tolerance checkpoint whose integrity is a critical determinant of TMZ sensitivity, particularly in GBM.

### 5.2. Non-Canonical MMR as TLS Facilitator

When canonical MMR signaling is impaired or attenuated, as frequently observed in recurrent GBM [13,15,19], non-canonical MMR functions may persist despite weakened mismatch recognition, creating a permissive state for TLS–mediated tolerance to TMZ-induced DNA lesions [67,83]. Reduced MutSα engagement, resulting from decreased MSH6 expression or altered MutSα complex stoichiometry, limits sustained recognition of O^6^-meG mismatches and diminish futile repair cycling, thereby shifting lesion processing toward DNA damage tolerance pathways [13,15,88]. Although O^6^-meG itself does not substantially impede replicative DNA synthesis [85,86], the relevant TLS substrate depends on MMR status. In MMR-proficient cells, RAD18-dependent TLS primarily fills MMR-generated ssDNA gaps following futile repair, whereas in MMR-hypomorphic cells, reduced formation of these intermediates favors direct bypass of unrepaired O^6^-meG at replication forks together with tolerance of other TMZ-induced lesions (N^7^-meG and N^3^-meA) and replication stress-associated structures [67,84]. Consequently, attenuation of MMR not only reduces cytotoxic checkpoint activation but also shifts the balance toward TLS-dependent lesion tolerance.

Residual non-canonical MMR activity may further promote this transition by modulating PCNA dynamics at stalled replication forks. Beyond their established role in mismatch recognition, MSH2 and MSH6 have been implicated in regulating PCNA retention and stability during genotoxic stress [89,90,91]. In TMZ-treated GBM cells, dysregulated MMR signaling is proposed to facilitate RAD6-RAD18-mediated PCNA monoubiquitination, the molecular switch that recruits specialized TLS polymerases [67,87]. Thus, rather than initiating canonical excision repair, non-canonical MMR activity may indirectly licence polymerase switching by reshaping the post-translational modification landscape of PCNA (Figure 3B). Defining the molecular events, and the contributions of individual MMR subcomplexes, that promote PCNA monoubiquitination will be critical for developing strategies to prevent TLS-mediated TMZ resistance [67].

Partial MMR attenuation also remodels replication checkpoint signaling. By reducing the generation of futile repair intermediates, hypomorphic MMR dampens ATR-CHK1 activation, shifting from the robust, acute checkpoint response characteristic of MMR-proficient cells to a lower-level, chronic signaling state that permits continued DNA replication under genotoxic stress [56,61,62,64,65]. This reduction in checkpoint enforcement lowers barriers to replication through damaged DNA and further favors TLS engagement. However, the relationship between checkpoint signaling and TMZ response appears to be context dependent [61,67]. Durable responses have been reported in models with compromised ATR signaling [92], raising the possibility that optimal TLS activation may require a threshold level of checkpoint signaling or, alternatively, that TLS can operate independently of ATR-CHK1 under specific conditions [61,67,83].

Collectively, these observations support a model in which hypomorphic MMR functions as a rheostat that balances cytotoxic signaling with DNA damage tolerance. Rather than representing a simple intermediate between MMR proficiency and complete deficiency, partial MMR attenuation creates a distinct adaptive state characterized by residual checkpoint signaling, enhanced TLS dependence, and increased mutagenic potential, thereby promoting cellular plasticity and therapeutic resistance [52,53,67]. This framework also provides mechanistic rationale for targeting TLS in MMR-hypomorphic tumors, where lesion tolerance remains dependent on residual MMR activity, while predicting diminished benefit in tumors with complete MMR loss [67,82].

### 5.3. TLS as an Evolutionary Engine in MMR-Altered GBM

Given its role in damage tolerance, TLS can be viewed as an adaptive mutagenic mechanism that becomes particularly consequential in tumors with altered MMR [17,67]. In normal cells, TLS enables replication across DNA lesions that would otherwise stall high-fidelity polymerases, albeit at the expense of reduced fidelity due to the lack of proofreading and relaxed base-pairing specificity of TLS polymerases [81,82]. In TMZ-treated cells, TLS acts on multiple substrates, including O^6^-meG—a miscoding but non-blocking lesion that can be bypassed by both replicative and TLS polymerases [85,86,93]—as well as the ssDNA gap intermediates generated during MMR processing of O^6^-meG mispairs and other replication stress-associated DNA structures arising from unrepaired alkylation damage [67]. Under MMR-proficient conditions, many TLS-induced misincorporations are corrected post-replicative MMR, thereby limiting the mutagenic consequences of lesion bypass [25,84]. In contrast, MMR deficiency or attenuation disrupts this proofreading function, allowing TLS-generated mismatches to become permanently fixed as mutations and converting TLS from a transient damage-tolerance mechanism into a major source of genetic diversification [17,18]. This results in increased mutation burden, including base substitutions and small insertions or deletions, particularly in genomic regions prone to replication stress [17,18].

This functional interplay between MMR and TLS has important evolutionary consequences during TMZ therapy. Persistent O^6^-meG lesions induce replication stress and promote TLS engagement. Persistent O^6^-meG lesions promote replication stress and TLS engagement [64,67]. Whereas MMR-proficient cells undergo futile repair cycles that activate checkpoint signaling and ultimately trigger growth arrest and cell death, MMR-deficient or MMR-hypomorphic cells increasingly rely on TLS-mediated lesion bypass to sustain DNA replication despite ongoing DNA damage [57,67]. Although this adaptive strategy promotes survival, it also accelerates the accumulation of base substitutions and small insertions or deletions, thereby increasing intratumoral heterogeneity and providing a substrate for clonal selection under therapeutic pressure [17,18,73]. Repeated TMZ exposure therefore couples replication stress tolerance with mutagenesis, driving the emergence of resistant subclones.

While the resulting hypermutator phenotype may increase neoantigen generation, its potential immunogenicity is often limited by the evolution of immune evasion mechanisms [17]. Thus, beyond facilitating lesion bypass, TLS functions as an evolutionary engine that promotes tumor plasticity and therapeutic resistance in MMR-altered GBM. These findings highlight TLS and its regulatory network as attractive therapeutic targets to constrain tumor evolution and potentially extend the efficacy of TMZ, particularly in MMR-hypomorphic tumors [67,82].

## 6. Clinical Correlates of MMR and TLS Status

### 6.1. Integrating MMR and TLS Status to Predict TMZ Response

A major limitation in predicting TMZ response in GBM is the continued reliance on binary biomarkers, particularly MGMT promoter methylation and MMR status [8,58]. Accumulating evidence suggests that this framework is insufficient to explain the marked heterogeneity in therapeutic response, especially among MGMT-methylated tumors [8,19,59]. An emerging paradigm posits that TMZ sensitivity is governed not by the presence or absence of MMR alone, but by the dynamic balance between MMR-mediated damage processing and TLS–mediated lesion tolerance [67]. Within this context, partial or hypomorphic MMR states, characterized by attenuated but not abolished repair activity, may play a central role in shaping therapeutic outcomes [52,59].

Defining partial MMR loss requires a shift toward functional assessment of pathway activity [58,59]. Unlike complete MMR deficiency, which is readily detected by MSI or loss of protein expression, hypomorphic states are often invisible to conventional diagnostics [58,59]. Functional assays, including plasmid-based MMR reporters and measurements of replication stress signaling (e.g., ATR–CHK1 activation, RPA accumulation), may provide a more direct readout of MMR proficiency along a continuum [94,95,96]. Integration of these approaches with proteomic analyses, such as the relative abundance and post-translational modification of MutSα versus MutSβ (MSH2–MSH3) complexes, may further reveal pathway rewiring that limits effective mismatch recognition and processing without complete loss of function [88]. These efforts can enable the development of an “MMR activity score” that more accurately reflects the tumor’s capacity to engage in cytotoxic futile repair following TMZ exposure [56,57].

In parallel, TLS pathway activation must be considered as a critical determinant of TMZ response [67,81]. TLS polymerases, including Pol η, Pol κ, Pol ι, and the REV1/Pol ζ complex, facilitate replication across damaged templates and mitigate replication fork collapse [81,82]. In the setting of partial MMR loss, reduced generation of futile repair intermediates and attenuated checkpoint signaling may lower the barrier for TLS engagement, thereby promoting lesion bypass and survival [64,67]. Functional assessment of TLS activity, through expression profiling, detection of PCNA monoubiquitination, and replication fork dynamics assays, could be integrated into a complementary “TLS activity score” [81,87]. Notably, TLS activation is not merely a passive backup mechanism but may represent an adaptive response that enables tumor cells to tolerate TMZ-induced lesions while accumulating mutations that drive clonal evolution and resistance [17,67].

The integration of MMR and TLS functional states provides a conceptual framework for predicting TMZ response based on the balance between cytotoxic processing and damage tolerance [67]. Tumors with intact MMR and low TLS activity are expected to exhibit robust TMZ sensitivity due to efficient induction of futile repair cycles and checkpoint-mediated apoptosis [56,57]. In contrast, tumors with profound MMR deficiency and high TLS activity are likely to be intrinsically resistant, relying on lesion bypass rather than damage signaling [59,67]. Importantly, an intermediate state characterized by partial MMR loss coupled with elevated TLS activity may define a clinically relevant subset of tumors with adaptive resistance, in which attenuated cytotoxic signaling is offset by enhanced tolerance mechanisms [52,67].

Translationally, this integrated model opens new avenues for biomarker development and therapeutic stratification [67]. Functional assays that simultaneously capture MMR and TLS activity, potentially in ex vivo tumor cultures or patient-derived organoids, could be combined with MGMT promoter methylation status to generate a composite predictive score [97,98,99]. Such an approach may refine patient selection for TMZ therapy and identify tumors that would benefit from rational combination strategies [99]. In particular, tumors exhibiting MMR-hypomorphic, TLS-dependent phenotypes may be uniquely vulnerable to therapeutic strategies that inhibit TLS pathways, thereby collapsing lesion tolerance and restoring TMZ sensitivity [82].

Collectively, these insights support a shift from static, single-pathway biomarkers toward an integrated, function-based framework in which TMZ response is defined by the interplay between DNA damage recognition, repair, and tolerance [59,67]. Prospective validation of this model in clinical cohorts will be essential to establish its predictive value and to guide the development of mechanism-based combination therapies for GBM [19,99].

### 6.2. Therapeutic Implications of Partial MMR Loss and TLS Activation in GBM

Recognition of partial, or hypomorphic MMR states alongside TLS activation has important therapeutic implications for GBM, particularly in the context of TMZ-based therapy [52,67]. Rather than a binary model of sensitivity versus resistance, these findings support a continuum in which treatment response is dictated by the balance between MMR-driven cytotoxic processing of O^6^-meG lesions and TLS-mediated lesion tolerance [59,67]. This conceptual shift provides a framework for rational therapeutic stratification and the design of mechanism-based combination strategies [67].

In tumors with intact MMR, TMZ induces robust futile repair cycling, leading to replication stress, checkpoint activation, and apoptotic cell death [56,57]. These tumors remain the most likely to benefit from TMZ monotherapy or standard chemoradiation [8]. In contrast, tumors with complete loss of MMR function, although relatively uncommon in primary GBM, exhibit primary resistance to TMZ due to failure to recognize and process O^6^-meG mismatches [13,15,59]. Such tumors may be better suited for alternative strategies that exploit their hypermutator phenotype, including immunotherapy-based approaches, although clinical efficacy in GBM remains variable [17].

The greatest therapeutic opportunity may lie within the intermediate, MMR-hypomorphic state [52,67]. In this setting, attenuated MMR activity reduces the formation of cytotoxic repair intermediates and dampens checkpoint signaling, thereby permitting survival in the presence of TMZ-induced DNA lesions [61,67]. Concomitant activation of TLS pathways further enhances this tolerance by enabling replication across damaged DNA [64,67]. This cooperative interaction may create a state of adaptive resistance, in which tumor cells evade TMZ-induced cytotoxicity while maintaining sufficient genomic plasticity to evolve under therapeutic pressure [17,67]. Importantly, this phenotype may be selectively targetable [67,82].

One promising strategy is the pharmacologic inhibition of TLS pathways to collapse lesion tolerance in MMR-hypomorphic tumors [82]. Disrupting key TLS components, such as REV1–Pol ζ interactions, polymerase recruitment to monoubiquitinated PCNA, or upstream regulators of TLS activation, may prevent bypass of TMZ-induced lesions, thereby restoring replication stress and re-engaging cell death pathways [82,87]. In this context, combining TMZ with TLS inhibitors represents a rational synthetic lethal approach, selectively targeting tumors that rely on TLS for survival under conditions of partial MMR loss [82]. To date, however, no TLS-targeted inhibitor has entered clinical evaluation in GBM, and current evidence derives largely from preclinical models [82].

An alternative, and potentially complementary, strategy is to modulate MMR activity or downstream checkpoint signaling [67]. For example, enhancing MMR function or stabilizing MutSα/MutLα complexes could increase the burden of futile repair intermediates, thereby amplifying TMZ cytotoxicity [55,57]. Conversely, targeting checkpoint adaptation pathways (e.g., ATR–CHK1 signaling) may sensitize tumors by preventing recovery from replication stress [36,61]. The therapeutic rationale for ATR-CHK1 inhibition differs by MMR context: in MMR-proficient tumors, ATR-CHK1 activation is already part of the cytotoxic cascade, and its inhibition may accelerate DNA damage accumulation and cell death [62]; in MMR-hypomorphic or -deficient tumors, where ATR-CHK1 signaling constitutes a survival dependency, checkpoint inhibition can induce synthetic lethality by abrogating the adaptive fork protection that enables tolerance of persistent alkylation damage [63,64,65]. However, the context-dependent roles of these pathways necessitate careful consideration, as excessive checkpoint inhibition in MMR-deficient settings may paradoxically promote tolerance or genomic instability [61,67].

These insights also have implications for treatment sequencing and biomarker-driven patient selection [59,67]. Functional stratification of tumors based on MMR and TLS activity could identify patients most likely to benefit from TMZ alone versus those requiring combination approaches [59,99]. For instance, patients with MMR-hypomorphic, TLS-high tumors may be prioritized for clinical trials combining TMZ with emerging TLS-targeted agents, whereas those with TLS-independent resistance mechanisms may require alternative therapeutic strategies [82]. Longitudinal monitoring of MMR and TLS states during treatment may further enable adaptive therapeutic interventions aimed at preventing or overcoming acquired resistance [19,73].

In summary, the intersection of partial MMR loss and TLS pathway activation defines a therapeutically actionable axis in GBM [67]. Exploiting this interplay through targeted inhibition of lesion tolerance mechanisms, combined with rational use of TMZ, may help overcome resistance and improve patient outcomes [82]. Future clinical studies integrating functional biomarkers with mechanism-based therapies will be critical to translate this conceptual framework into effective treatment strategies [19,99].

## 7. Strategies to Delay or Bypass MMR-Mediated Resistance

TMZ resistance in GBM is frequently shaped by alterations in MMR function, ranging from complete loss of key components such as MSH6 to more subtle, hypomorphic states that attenuate cytotoxic signaling [17,36]. These adaptations diminish the formation of futile repair intermediates and weaken checkpoint activation, thereby enabling tumor cells to tolerate O^6^-meG lesions [36,64]. Overcoming MMR-mediated resistance, therefore, requires strategies that either restore cytotoxic processing of DNA damage or eliminate compensatory tolerance mechanisms that sustain tumor survival. Several promising strategies are under investigation, although challenges remain that limit their immediate clinical translation.

### 7.1. Targeting Compensatory Repair Pathways

In MMR-deficient GBM, TMZ resistance arises from impaired processing of O^6^-meG lesions, which prevents futile repair cycling and downstream cytotoxic signaling [36,100]. As a result, tumor cells increasingly rely on compensatory DNA damage tolerance pathways to replicate despite alkylation-induced lesions. One key mechanism involves TLS, where specialized polymerases, including REV1 and Pol ζ, bypass TMZ-induced lesions to sustain DNA replication [81,82,87]. Targeting TLS components or regulators of PCNA monoubiquitination can block lesion bypass and convert a tolerant phenotype into lethal replication stress, particularly in MMR-hypomorphic tumors in which TLS appears to be especially important for survival [64,82,87].

A complementary strategy focuses on the replication stress and checkpoint dependencies that emerge in MMR-deficient or hypomorphic tumors. Reduced MMR activity alters the dynamics of replication fork progression and DNA damage signaling, often creating reliance on ATR–CHK1–mediated checkpoint pathways for survival [63,64,65]. Pharmacologic inhibition of ATR, CHK1, or downstream effectors can abrogate this adaptive response, driving replication catastrophe and cell death (Table 1) [101,102,103]. Notably, the effectiveness of checkpoint inhibition may depend on the degree of residual MMR activity, underscoring the importance of functional stratification [63,65].

An emerging strategy to overcome TMZ resistance in MMR-deficient GBM is synthetic lethality through DNA polymerase theta (POLθ) inhibition. POLθ mediates microhomology-mediated end joining, an error-prone compensatory repair pathway on which cells with defective high-fidelity repair can become heavily dependent [104,105]. In such contexts, POLθ contributes to the resolution of double-strand breaks and collapsed replication forks, including those that may arise from unresolved O^6^-meG lesions [104,106,107,108]. Inhibiting POLθ selectively compromises MMR-deficient cells by exacerbating DNA damage and genomic instability, a vulnerability further amplified in combination with TMZ [104,105,107,108]. Although direct evidence in GBM remains limited, targeting POLθ represents a rational approach to preferentially eliminate MMR-deficient tumor cells while sparing MMR-proficient cells, and it warrants further preclinical and clinical evaluation.

PARP inhibitors represent an additional avenue for exploiting DNA repair deficiencies, acting primarily by trapping PARP on DNA and exacerbating replication stress [36,100]. While single-agent PARP inhibition shows limited efficacy in MMR-deficient GBM—likely due in part to elevated HR- and TLS-mediated lesion tolerance—combining PARP inhibitors with DNA-damaging agents such as TMZ or radiotherapy can overwhelm residual repair capacity, induce replication stress, and sensitize tumors to cytotoxicity [36,109,110]. Consistent with this, preclinical GBM studies indicate that PARP-inhibitor sensitization is driven by replication stress, is associated with MGMT promoter methylation as a candidate biomarker and is constrained by blood–brain barrier penetration and intratumoral heterogeneity (Table 1) [111,112,113]. Furthermore, because PARP inhibitor sensitization may depend in part on intact processing of replication-associated DNA lesions, its therapeutic benefit is likely diminished in tumors with profound MMR deficiency. Whether hypomorphic MMR states confer increased susceptibility to PARP inhibitor-based combinations and can serve as predictive biomarkers remains an important area for future investigation.

Collectively, these strategies illustrate a framework in which synthetic lethality, TLS blockade, checkpoint inhibition, and POLθ or PARP targeting can be rationally integrated to counter intrinsic and adaptive TMZ resistance in MMR-compromised GBM, supporting precision-guided therapeutic interventions [36,100].

### 7.2. Epigenetic Modulation

Epigenetic modulation represents a tractable avenue to exploit MMR dysfunction in GBM, particularly in tumors exhibiting partial or context-dependent loss of MMR activity. Beyond genetic alterations, MMR components are frequently regulated by promoter methylation, chromatin accessibility, and post-translational modifications that influence complex assembly and function [114,115]. Therapeutically, this raises the possibility of modulating MMR proficiency to alter TMZ response. For example, DNA methyltransferase inhibitors can restore expression of MMR proteins such as MLH1, thereby enhancing futile repair cycling and sensitizing tumors to TMZ [114]; histone deacetylase inhibition has also been proposed to restore MSH6 expression, though this remains to be formally demonstrated [116]. Conversely, where excessive repair capacity promotes tolerance through pathway rewiring, targeted epigenetic modulation might disrupt compensatory chromatin states that facilitate lesion bypass and replication stress adaptation, including those that favor TLS engagement [115]. Epigenetic therapies may also reshape the broader DDR landscape—for instance, by modulating chromatin accessibility at stalled forks, altering checkpoint signaling thresholds, and influencing mutational trajectories [115,117]. Integrating epigenetic profiling with functional MMR and TLS readouts may, therefore, enable biomarker-driven deployment of epigenetic agents, either to restore TMZ sensitivity or to potentiate combination strategies that target lesion tolerance pathways in MMR-hypomorphic GBM [114,117].

### 7.3. Immunotherapy Exploitation

Beyond its effects on DNA repair, MMR dysfunction, particularly in its partial or evolving forms, can reshape tumor immunogenicity in ways that may be therapeutically relevant in GBM. Impaired MMR activity promotes the accumulation of mutations, including frameshift and base substitution events, which can generate neoantigens capable of eliciting antitumor immune responses [17]. However, classical hypermutation and MSI are uncommon in GBM. Even submaximal increases in mutational burden arising from MMR attenuation, especially under TMZ pressure, have not reliably translated into enhanced tumor immune recognition, as tumor mutation burden correlates poorly with checkpoint–blockade response in glioma [17,118,119,120]. In parallel, persistent DNA damage and replication stress associated with defective MMR can, at least preclinically, lead to cytosolic DNA accumulation and activation of innate immune pathways such as cGAS–STING, promoting type I interferon signaling and immune priming [18,65]. These observations have motivated strategies that combine TMZ with immune checkpoint blockade or innate immune agonists. To date, however, somatic MMR-deficient and TMZ-induced hypermutant GBM have generally not responded to checkpoint-blockade (Table 1), reflecting the profoundly immunosuppressive brain tumor microenvironment and PD1/PD-L1-independent immune evasion, whereas durable responses have been observed mainly in tumors arising from germline biallelic MMR-deficiency [121,122,123]. Careful temporal integration may be required, as excessive immunoediting or T-cell exhaustion could limit durable responses [122,123]. Thus, leveraging the immunologic sequelae of MMR dysfunction remains a context-dependent and largely investigational avenue in GBM rather than an established therapeutic strategy [18,118].

**Table 1:** Therapeutic strategies for MMR-deficient glioblastoma: alkylator-based and immunotherapy approaches.

Strategy	Agent(s)	Model/Population	MMR Status	Key Findings	Stage/Outcome	Reference(s)
Alkylator and DNA Damage-Based Strategies						
Nitrosourea salvage therapy	Lomustine (CCNU), Nimustine (ACNU)	TMZ-resistant GBM cell lines and xenografts	Acquired TMZ resistance (MMR-related)	CCNU and ACNU retained antitumor activity in TMZ-resistant GBM; both induced apoptosis despite established TMZ resistance; ↑ survival in xenograft-bearing mice	Preclinical	[124]
DNA cross-linking imidazotetrazine	KL-50	Patient-derived GBM, recurrent post-TMZ models, MSH6-KO GBM; intracranial PDX (GBM6, GBM12)	MMR-proficient and MMR-deficient (MSH6-deficient)	KL-50 generates cytotoxic DNA interstrand crosslinks independently of MMR; ↑ survival in both treatment-naïve and recurrent post-TMZ models regardless of MMR status	Preclinical	[123]
TMZ rechallenge	TMZ retreatment	Recurrent hypermutant gliomas	Acquired MMR deficiency	Generally ineffective; MMR loss abolishes futile-cycle cytotoxicity that mediates TMZ response	Clinical	[17]
TMZ + PARP inhibition	Veliparib, Pamiparib, Olaparib, Niraparib	GBM preclinical models	Not restricted to MMR-deficient tumors	PARP inhibition ↑ TMZ-induced replication stress and DNA damage; potential utility before complete MMR loss; efficacy after established MMR deficiency uncertain.	Preclinical/Clinical	[36,125]
TMZ + ATR inhibition	ATR inhibitors (experimental)	Hypermutant/MMR-altered glioma models	MMR-impaired	ATR dependency emerges following MMR attenuation and replication stress adaptation; proposed strategy to prevent lesion tolerance	Preclinical	[126]
TMZ + TLS pathway inhibition	REV1, RAD18, Polζ targeting (experimental)	Hypermutant/TMZ-resistant models	MMR-deficient or MMR-attenuated	TLS facilitates O^6^-meG tolerance after MMR attenuation; inhibition may restore alkylator sensitivity	Preclinical	[17,126]
Immunotherapy-Based Strategies						
PD-1 blockade	Pembrolizumab, Nivolumab	Recurrent hypermutant glioma patients; 11 MMR-deficient glioma patients (retrospective)	Acquired MMR deficiency (primarily MSH6-mutant); de novo and treatment-induced	Initial rationale based on ↑ TMB and predicted neoantigen burden; however, most patients exhibited progressive disease; limited T-cell infiltration observed	Clinical—limited efficacy (median PFS ~1.4 mo; 82% progressed as best response)	[17]
PD-1 + CTLA-4 blockade	Anti-PD-1 + Anti-CTLA-4	Syngeneic hypermutated orthotopic glioma model; experimental hypermutant gliomas	Hypermutant/MMR-deficient-like	Combination therapy suppressed tumor growth and produced durable responses in a subset; greater efficacy than PD-1 alone; however, significant heterogeneity with responder and non-responder populations despite identical genetics	Preclinical—incomplete and variable responses	[127]
Macrophage-directed immunotherapy + ICB	PD-L1 targeting on TAMs	Hypermutated glioma mouse models	Hypermutant/MMR-deficient-like	Resistant tumors exhibited enrichment of PD-L1^+^ macrophages; TAM-directed therapy may overcome resistance to checkpoint blockade	Preclinical	[127]
Neoantigen-driven immunotherapy	Vaccines/adoptive T-cell approaches	Hypermutant gliomas	MMR-deficient	↑ Mutational burden theoretically ↑ neoantigen repertoire; however, high intratumoral heterogeneity and subclonality likely limit efficacy	Conceptual	[17]
Hypermutation-guided immunotherapy	Immune checkpoint inhibitors (broadly)	Hypermutant high-grade gliomas	MMR-deficient	High TMB initially proposed as predictive biomarker; subsequent studies concluded hypermutation alone is insufficient to predict benefit	Clinical—hypermutation alone not predictive	[128]

MMR = mismatch repair; GBM = glioblastoma; TMZ = temozolomide; CCNU = lomustine; ACNU = nimustine; PDX = patient-derived xenograft; KO = knockout; PARP = poly(ADP-ribose) polymerase; ATR = ataxia telangiectasia and Rad3-related kinase; TLS = translesion synthesis; Polζ = DNA polymerase zeta; O6-meG = O6-methylguanine; PD-1 = programmed cell death protein 1; CTLA-4 = cytotoxic T-lymphocyte-associated protein 4; ICB = immune checkpoint blockade; TAM = tumor-associated macrophage; TMB = tumor mutational burden; PFS = progression-free survival; ↑ = increase/induce. Section color coding: ■ Alkylator/DNA damage ■ Immunotherapy.

### 7.4. Alternative Alkylators and Combination Regimens

An additional therapeutic strategy for TMZ-resistant GBM involves the use of alternative alkylating agents and rational combination regimens designed to bypass or exploit defects in DNA repair pathways (Table 1). Agents such as lomustine (CCNU), KL-50, or other nitrosoureas generate a broader spectrum of DNA lesions, including interstrand crosslinks and N7-guanine adducts—that are less dependent on O^6^-meG processing and may retain efficacy in tumors with attenuated MMR [129,130,131]. In particular, KL-50 has been designed to form interstrand crosslinks in an MMR independent manner that is selective for MGMT-deficient cells [130,132,133]. In parallel, combination approaches aim to enhance cytotoxicity either by increasing lesion burden or by disabling compensatory repair and tolerance mechanisms. For example, co-administration of alkylators with PARP inhibitors can potentiate DNA damage accumulation, while integration with radiotherapy amplifies replication stress and DNA strand break formation [109,134]. In the context of partial MMR loss and TLS activation, such regimens may be particularly effective when paired with agents that suppress lesion bypass or replication fork recovery, thereby shifting the balance from tolerance toward cytotoxicity [100,134]. Collectively, these strategies underscore the potential of diversifying alkylator-based therapy and leveraging combination treatments to overcome resistance and extend the therapeutic benefit of DNA-damaging agents in GBM [129,135].

Collectively, these strategies highlight the need for a multifaceted approach to overcome MMR-mediated resistance in GBM. Integrating functional assessment of MMR activity with characterization of compensatory pathways such as TLS and replication stress signaling will be essential for guiding therapeutic selection [36,100]. Ultimately, rational combination therapies that exploit the specific vulnerabilities created by MMR dysfunction hold the considerable promise for improving outcomes in this challenging disease [36].

## 8. Future Perspectives

A key priority moving forward is to move beyond the binary classification of MMR as “proficient” or “deficient” and instead establish a quantitative framework that captures partial or hypomorphic MMR activity [19,58,59]. In the context of TMZ response in GBM, subtle reductions in MMR efficiency, rather than complete loss, may critically determine whether O^6^-meG lesions undergo cytotoxic futile repair or are diverted toward DNA damage tolerance pathways [52,57,67]. Defining this intermediate state will require functional, rather than purely genomic, metrics [13,15]. Emerging approaches, including plasmid-based MMR reporter assays [94,95,96], single-cell replication stress profiling [136], and dynamic assessment of ATR–CHK1 signaling [56,61] could be integrated into an “MMR activity score” that quantitatively reflects repair capacity. Complementing these assays with proteomic analyses of post-translational modifications and complex stoichiometry (e.g., MutSα vs. MutSβ balance) may further refine our understanding of how MMR is rewired in TMZ-treated tumors [88].

A second priority is to define how partial MMR loss reshapes TLS pathway utilization. We propose that attenuated MMR lowers the threshold for TLS engagement by reducing the futile repair intermediates and checkpoint activation, thereby promoting lesion bypass by specialized polymerases such as Pol η, Pol κ, Pol ι, REV1/Pol ζ to bypass O^6^-meG lesions [64,81,82,87]. Conversely, accumulating evidence suggests that dysregulated TLS can also influence MMR activity; for example, RAD18-MSH2 interactions may redirect MMR toward lesion tolerance [67,83]. Thus, the MMR–TLS relationship is likely bidirectional, with TLS functioning as an adaptive replication stress response rather than merely a backup pathway. Future studies employing high-resolution approaches such as isolation of proteins on nascent DNA (iPOND) [137], single-molecule DNA fiber assays [138], and live-cell imaging of polymerase dynamics [139] should help define the temporal and spatial coordination of these pathways at stalled replication forks.

An emerging area deserving greater attention is the role of metabolic adaptation in regulating the MMR–TLS transition. Enhanced pentose phosphate pathway activity and RRM2-dependent dNTP synthesis support TLS-mediated lesion tolerance by sustaining replication under stress [140,141], whereas nucleotide depletion favors MMR-dependent ssDNA gap accumulation and cytotoxicity [57]. Likewise, PARP1-mediated NAD^+^ consumption during MMR processing links repair pathway choice to cellular bioenergetics and redox homeostasis [74]. These observations suggest that metabolic rewiring acts as a replication stress checkpoint that influences whether damaged cells undergo MMR-mediated cytotoxicity or TLS-dependent survival, highlighting nucleotide metabolism and cellular bioenergetics as potential therapeutic targets to enhance TMZ efficacy [76,141].

These mechanistic insights have important translational implications. Tumors with intact MMR are generally TMZ-sensitive, whereas complete MMR deficiency confers primary resistance while creating alternative therapeutic opportunities, including increased susceptibility to immune checkpoint blockade in hypermutated tumors [17,118,121,122]. The intermediate, MMR-hypomorphic state may represent a particularly actionable therapeutic window, as these tumors retain sufficient repair activity to avoid catastrophic genomic instability but become increasingly dependent on TLS for survival under TMZ-induced replication stress [52,67]. This raises the possibility of synthetic lethal strategies combining TMZ with inhibitors targeting the REV1–Pol ζ axis or other TLS components, although these approaches remain at preclinical stage [81,82].

Ultimately, realizing precision TMZ therapy will require prospective clinical studies that integrate functional MMR assays, TLS biomarkers, metabolic profiling, and MGMT promoter methylation into patient stratification [8,19,94]. Rather than viewing TMZ response as a static property dictated by a single biomarker, future therapeutic strategies should consider it as a dynamic phenotype governed by the interplay among MMR activity, replication stress signaling, TLS-mediated damage tolerance, and metabolic adaptation.

## 9. Summary and Conclusions

Canonical and non-canonical MMR represent distinct biological programs with opposing consequences for TMZ response: canonical MMR enforces lesion-induced cytotoxicity, whereas non-canonical MMR promotes stress adaptation, lesion tolerance, and evolutionary escape [52,57,67]. Critically, TMZ resistance does not require complete loss of MMR; GBM can exploit intermediate, hypomorphic states, which is why a binary proficient/deficient classification is insufficient [19,58,59].

This framework exposes at least three therapeutic vulnerabilities. First, MMR–TLS crosstalk can elevate tumor mutational burden and neoantigens, conferring responsiveness to immune checkpoint blockade when the underlying mutational processes remain active and immunogenic [17,118,121,122]. Second, chronic reliance on TLS also reprograms replication-stress responses and may compromise high-fidelity repair pathways such as HR [104,106,142]; this creates synthetic vulnerabilities to PARP inhibitors and supports combining TMZ with PARP inhibition, particularly in tumors with persistent PCNA monoubiquitination and active polymerase switch, as a potential biomarker-guided precision therapy for GBM [36,110,143,144]. Third, MMR-deficient tumors show heightened sensitivity to DNA crosslinking agents such as CCNU (lomustine) and KL-50 [130,131,132,133,135]; similar mechanisms likely operate in tumors with non-canonical MMR activity, broadening the therapeutic utility of DNA crosslinkers such as KL-50.

More broadly, non-canonical MMR activity imposes a set of collateral dependencies—on immune surveillance, replication stress tolerance, and the wider DNA-damage response networks—that can be exploited therapeutically [36,67,104]. Defining these dependencies should enable rational combination strategies designed to counteract TLS-driven TMZ resistance and to convert adaptive flexibility of GBM into a therapeutic liability [81,82,133].

## 10. Methods

This expert review was conducted as a narrative synthesis rather than a systematic review or meta-analysis, reflecting existing knowledge gaps in temozolomide (TMZ) sensitivity and resistance, particularly at the intersection of MMR, TLS, and replication stress. A systematic approach with rigid inclusion criteria would likely fail to capture the conceptual breadth and emerging paradigm of a “MMR–TLS hypomorphic state” potentially driving progressive hypermutation and therapeutic resistance. Relevant studies were identified through PubMed search using combinations of keywords, including “mismatch repair,” “Translesion synthesis,” “temozolomide resistance,” “glioblastoma therapy response,” “DNA damage response,” and “replication stress,” combined using Boolean operators (“AND,” “OR”). Reference lists of selected publications were additionally screened to identify relevant publications not captured during the initial database search. Inclusion criteria comprised original experimental investigations, clinical studies, and review articles focused on relevant topics covered in this article. Exclusion criteria included case reports, editorials, and studies focusing on TMZ resistance mechanisms other than DDR or replication pathways. The primary search window spanned 2015–2026, with earlier studies incorporated where necessary to address foundational or underexplored areas.

## Data Availability

No new data were created or analyzed in this study. Data sharing is not applicable to this article.

## Abbreviations

The following abbreviations are used in this manuscript:

ATM	Ataxia-Telangiectasia Mutated
ATR	Ataxia Telangiectasia and Rad3-Related
CHK1	Checkpoint Kinase 1
CLIA	Clinical Laboratory Improvement Amendments
cGAS	Cyclic GMP-AMP Synthase
DNA	Deoxyribonucleic Acid
MLH1	MutL homolog 1
MSH2	MutS homolog 2
MSH6	MutS homolog 6
PMS2	PMS1 protein homolog 2
NAD^+^	Nicotinamide Adenine Dinucleotide
PCNA	Proliferating Cell Nuclear Antigen
STING	Stimulator of Interferon Genes
SETD2	SET domain containing 2
RAD6	Radiation-sensitive 6 protein
RAD18	E3 ubiquitin protein ligase RAD18
